# Abatacept Rescue Therapy in Kidney Transplant Recipients: A Case Series of Five Patients

**DOI:** 10.3389/ti.2022.10681

**Published:** 2022-08-12

**Authors:** Charlotte Uro-Coste, Alba Atenza, Anne-Elisabeth Heng, Paul-Olivier Rouzaire, Cyril Garrouste

**Affiliations:** ^1^ Department of Nephrology, Dialysis and Transplantation, Clermont-Ferrand, France; ^2^ EA 7453 CHELTER, Clermont-Ferrand, France; ^3^ Histocompatibility and Immunogenetics Laboratory, Clermont-Ferrand, France

**Keywords:** belatacept conversion, abatacept, kidney transplant, CMV infection, immunosuppression, rescue therapy

Dear Editors,

Abatacept, a cytotoxic T-lymphocyte-associated antigen 4 immunoglobulin (CTLA4-Ig), is a subcutaneously administered immunosuppressive drug that selectively inhibits T-cell activation by blocking the CD28-CD80/86 costimulatory pathway. Abatacept is widely used in rheumatology, especially in the treatment of rheumatoid arthritis (1). More recently, intravenously administered belatacept, another CTLA4-Ig, has shown better renal transplant (RT) survival results, improvement in long-term renal function, and less *de novo* donor-specific antibody (DSA) formation than a calcineurin inhibitor (CNI) regimen, either in induction therapy or after conversion from CNIs (2–4). However, to date, abatacept has been reported only exceptionally as maintenance treatment in patients who have undergone renal transplantation (5).

This letter reports on our experience with CNI conversion to self-administered subcutaneous abatacept in five patients who benefited from RT for 1.5–84 months (Table 1). The initiation of CTLA4-Ig therapy was motivated by graft biopsy-confirmed CNI toxicity in four patients (P1, P2, P3, and P4) and varying concentrations of tacrolimus owing to severe gastroparesis (P5). Abatacept maintenance therapy was chosen due to difficult peripheral venous access or to avoid hospitalization in the context of the COVID-19 pandemic. All patients received a 125 mg subcutaneous injection of abatacept every week (6). The first injection was performed in Day Hospital for monitoring and injection education. Treatment with CNIs was progressively withdrawn over 1–3 months (7). All patients received prednisone 5 mg/day and mycophenolate mofetil (P1, P2, P4, and P5) or everolimus (P3).

**TABLE 1:** Patient characteristics, conversion and follow-up data.

		Patient 1	Patient 2	Patient 3	Patient 4	Patient 5
Patients’ characteristics	Age at Tx (y)	76	47	67	61	26
	Sex	M	M	M	M	F
	Weight (kg)	60	98	89	80	57
	ESRD diagnosis	Diabetes	APLS	Glomerulonephritis	IgA nephropathy	Lupus
Transplant characteristics	Re Tx	No	No	No	No	Yes
	Induction therapy	Antithymocyte globulin	Basiliximab	Basiliximab	Basiliximab	Antithymocyte globulin
	CMV status	D+/R+	D+/R−	D+/R−	D−/R+	D−/R+
Switch data	Conversion indication	CNI toxicity	CNI toxicity	CNI toxicity	CNI toxicity	gastroparesis
	Reason for choosing abatacept	COVID-19 pandemic	Difficult venous access	Difficult venous access	Difficult venous access	Difficult venous access
	Time of conversion post-Tx (m)	1,5	84	32	84	13
	Associated treatment	MPA	MPA	Everolimus	MPA	MPA
Complication	Rejection	No	No	No	No	No
	Viral complication	CMV disease at M5	No	No	No	CMV disease at M3
Creatinine (µmol/L)/eGFR MDRD (ml/min/1.73 m^2^)	Month -1	193/31	266/23	263/22	309/19	181/31
	Month 0	190/32	258/24	230/26	320/18	172/33
	Month 1	175/35	270/23	271/22	272/22	170/33
	Month 2	168/37	303/20	270/22	322/18	187/30
	Month 3	181/34	236/27	279/21	289/20	191/29
	Month 6	213/28	238/26	269/22	295/20	188/30
	Last follow-up	194/31	238/26	256/23	303/19	169/33
DSA follow-up	DSA at switch	Yes (score 4)	No	No	No	No
	Last DSA	Neg	Neg	Neg	Neg	Neg
Time on abatacept (m)		5	6	16	9	17

APLS, antiphospholipid syndrome; CNI, calcineurin inhibitor; D, donor; DSA, donor-specific antibody; eGFR, estimated glomerular function rate; ESRD, end‐stage renal disease; kg, kilogram; m, month; MPA, mycophenolic acid; R, recipient; Tx, transplant; y, year.

The mean follow-up after switching to abatacept was 13.6 months. In all patients, renal function was similar between baseline and the last follow-up (Table 1). We did not observe any transplant rejection or any appearance of or increase in DSAs, which were routinely screened every 3 months (screening and single antigen identification, One Lambda Thermo Fisher). Two patients developed CMV disease (P1 and P5). It is of note that P5 was not receiving any CMV prophylaxis. In P1, CMV infection was refractory to available antivirals (valganciclovir, foscarnet) and the discontinuation of abatacept. Treatment with maribavir for 8 weeks reduced the viral load to less than 2,000 IU/ml, and viral load remained stable with only azathioprine and prednisone. In addition, we observed better control of blood pressure in P2 and P3, allowing the cessation of some of the antihypertensive drugs.

In RT recipients with CNI intolerance, conversion to belatacept is an effective and validated option. However, this treatment has logistical drawbacks due to its intravenous formulation and its nurse-supervised infusion for 30–60 min (8). In patients with rheumatoid arthritis, a fixed-dose SC administration of 125 mg weekly compared with the body-weight-based monthly IV administration of 10 mg/kg allowed to obtain therapeutic concentrations and similar clinical remission rates irrespective of baseline patient body mass index (6).

In the literature, only one case series reported nine adult RT recipients who received abatacept due to CNI intolerance and belatacept unavailability (5). In this cohort, patients received abatacept at approximately 10 mg/kg, mainly intravenously (*N* = 8), for a median duration of 82 months, and the authors reported stable long‐term RT function, even though one patient developed a grade 1A acute cellular rejection episode with a favorable outcome.

In our cohort of patients treated subcutaneously with a fixed weekly dose of 125 mg, irrespective of the patient weight (from 1.27 to 2.19 mg/kg), no transplant rejection or DSA appearance was observed. Even though abatacept is known to display lower binding avidity to CD80 and CD86 than belatacept (9), these encouraging data are consistent with the fact that CD86 occupancy in belatacept-treated kidney transplant patients seems not to be associated with clinical and infectious outcomes.

Special attention should, however, be paid to the occurrence of opportunistic infections and their prophylactic treatments (10). Indeed, CMV infection is a frequently reported complication after conversion to belatacept, especially in the first 6 months after transplantation in elderly patients and patients with an estimated glomerular filtration rate <25/ml/min/1.73 m^2^ (10).

In conclusion, our local experience suggests that weekly subcutaneous administration of 125 mg abatacept may be an effective alternative to belatacept, with a similar safety profile, as rescue therapy in RT recipients with peripheral difficult venous access and/or wishing to be more autonomous. These exciting findings need to be confirmed by further larger, prospective, and randomized studies.

## Data Availability

The original contributions presented in the study are included in the article/supplementary material, further inquiries can be directed to the corresponding author.
